# Supplementary figures and images for: Development and validation of a cellular host response test as an early diagnostic for sepsis
Source: PLoS One. 2021 Apr 15;16(4):e0246980. doi: 10.1371/journal.pone.0246980 (PMC8049231; doi:10.1371/journal.pone.0246980)

**
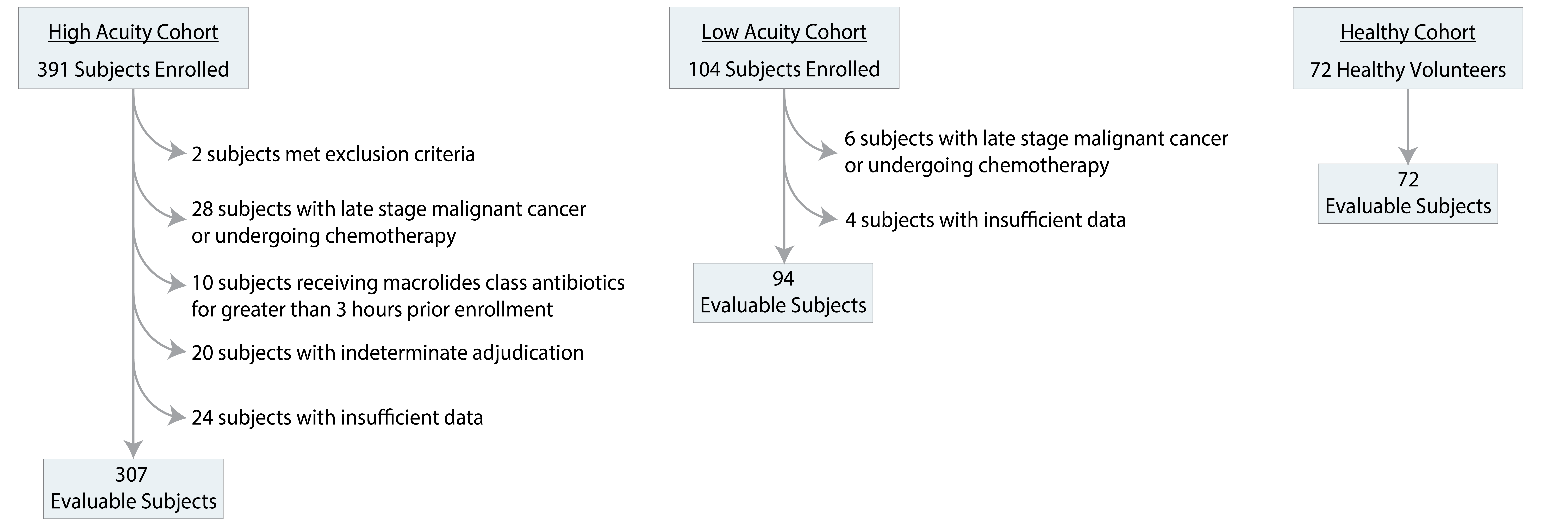
**

**S1 Fig. Flow chart for selection of evaluable subjects and exclusion of ineligible subjects.**

Supplement: S1 Fig — (DOCX) [file pone.0246980.s001.docx]
