# Supplementary material for: Development and validation of a cellular host response test as an early diagnostic for sepsis
Source: PLoS One. 2021 Apr 15;16(4):e0246980. doi: 10.1371/journal.pone.0246980 (PMC8049231; doi:10.1371/journal.pone.0246980)
Supplement: S2 Fig — (DOCX) [file pone.0246980.s002.docx]

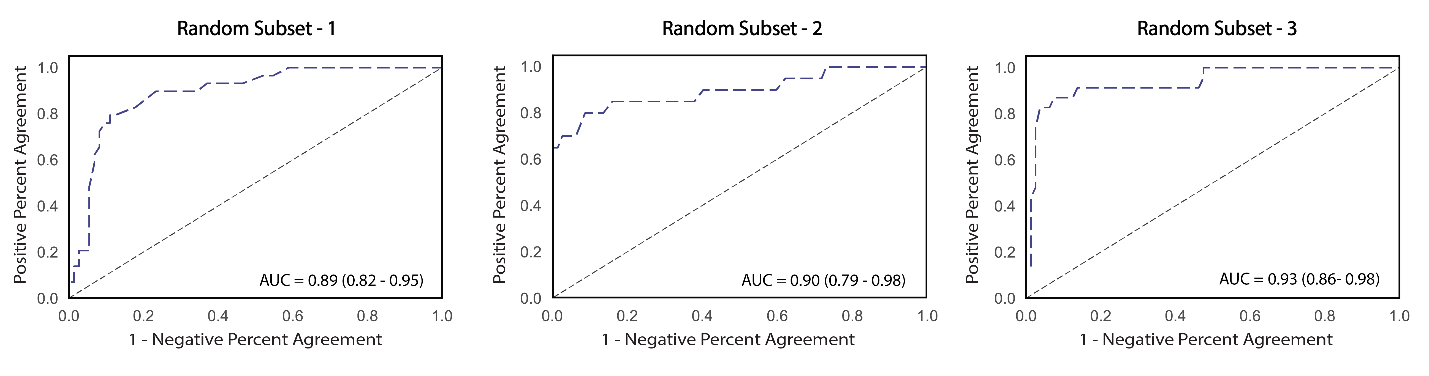


**S2 Fig. Receiver Operating Characteristic (ROC) curves showing the 3-fold cross-validation of the high acuity cohort.**
