# Supplementary material for: Development and validation of a cellular host response test as an early diagnostic for sepsis
Source: PLoS One. 2021 Apr 15;16(4):e0246980. doi: 10.1371/journal.pone.0246980 (PMC8049231; doi:10.1371/journal.pone.0246980)
Supplement: S3 Fig — Dotted black line indicates the mean AUC of 0.91. (DOCX) [file pone.0246980.s003.docx]

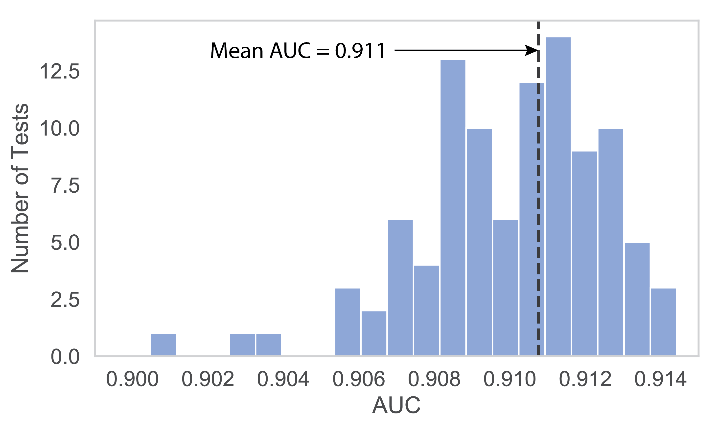


**S3 Fig. Distribution of cross-validated AUC for repeated 10-fold partitions of the high acuity cohort.** Dotted black line indicates the mean AUC of 0.91.
