# Supplementary material for: Development and validation of a cellular host response test as an early diagnostic for sepsis
Source: PLoS One. 2021 Apr 15;16(4):e0246980. doi: 10.1371/journal.pone.0246980 (PMC8049231; doi:10.1371/journal.pone.0246980)
Supplement: S1 Table — (DOCX) [file pone.0246980.s004.docx]

**S1 Table. Effect size, as measured by Cohen’s d, for data in Fig 2.**

| **Cell type** | **Mean Aspect Ratio** | | | **Mean VEIR** | | |
| --- | --- | --- | --- | --- | --- | --- |
|  | **Healthy vs. SIRS 2+** | **SIRS 2+ vs. Septic** | **Healthy vs. Septic** | **Healthy vs. SIRS 2+** | **SIRS 2+ vs. Septic** | **Healthy vs. Septic** |
| Lymphocytes | 0.87 | -0.40 | 0.46 | -0.91 | 0.52 | -0.35 |
| Neutrophils | -1.16 | -0.68 | -1.99 | -0.71 | -1.43 | -1.90 |
| Monocytes | -0.22 | -0.54 | -0.77 | -0.79 | -1.42 | -2.50 |

VEIR, visco-elastic inertial response
