# Supplementary material for: Development and validation of a cellular host response test as an early diagnostic for sepsis
Source: PLoS One. 2021 Apr 15;16(4):e0246980. doi: 10.1371/journal.pone.0246980 (PMC8049231; doi:10.1371/journal.pone.0246980)
Supplement: S2 Table — (DOCX) [file pone.0246980.s005.docx]

**S2 Table. Baseline demographics (age, sex, and race) across interpretation bands for subjects in each of the three cohorts.**

| **Characteristic** | | **Cohort** | **Green**  **Band** | **Yellow Band** | **Red**  **Band** |
| --- | --- | --- | --- | --- | --- |
| Age, median (IQR) | | High Acuity | 58.7 (46-71) | 52.0 (63-77) | 57.0 (56-85) |
| Gender (Female), N (%) | |  | 96 (49.2) | 29 (54.7) | 29 (49.2) |
| Race, N (%) | White |  | 94 (48.2) | 40 (75.4) | 29 (49.2) |
|  | African American |  | 88 (45.1) | 10 (18.9) | 25 (42.4) |
|  | Other |  | 13 (6.7) | 3 (5.7) | 5 (8.5) |
| Age, median (IQR) | | Low Acuity | 54.4 (37-71) | 60.5 (51-64) | 61.9 (54-70) |
| Gender (Female), N (%) | |  | 33 (45.2) | 4 (40.0) | 7 (63.6) |
| Race, N (%) | White |  | 34 (46.6) | 6 (60.0) | 7 (63.6) |
|  | African American |  | 36 (49.3) | 4 (40.0) | 4 (36.4) |
|  | Other |  | 3 (4.1) | 0 (0.0) | 0 (0.0) |
| Age, median (IQR) | | Healthy | 52.5 (38-68) | 0 (0.0) | 0 (0.0) |
| Gender (Female), N (%) | |  | 40 (55.6) | 0 (0.0) | 0 (0.0) |
| Race, N (%) | White |  | 63 (87.5) | 0 (0.0) | 0 (0.0) |
|  | African American |  | 4 (5.6) | 0 (0.0) | 0 (0.0) |
|  | Other |  | 3 (4.2) | 0 (0.0) | 0 (0.0) |

Abbreviations: IQR, interquartile range (Q1 – Q3).
